# Green Synthesis of Novel Polyaniline Nanofibers: Application in pH Sensing

**DOI:** 10.3390/molecules201018585

**Published:** 2015-10-13

**Authors:** Shivani Tanwar, Ja-an Annie Ho

**Affiliations:** BioAnalytical Chemistry and NanoBiomedicine Laboratory, Department of Biochemical Science and Technology, National Taiwan University, No. 1, Sec. 4, Roosevelt Road, Taipei 10617, Taiwan; E-Mail: tanwarshivani@gmail.com

**Keywords:** chiral polyaniline, nanofiber, synthesis, pH sensor, absorption spectroscopy

## Abstract

An optically active polyaniline nanomaterial (PANI-Nap), doped with (*S*)-naproxen, was developed and evaluated as a potent pH sensor. We synthesized the material in one pot by the addition of the dopant, (*S*)-naproxen, prior to polymerization, followed by the addition of the oxidizing agent (ammonium persulfate) that causes polymerization of the aniline. This green chemistry approach allowed us to take only 1 h to produce a water-soluble and stable nanomaterial. UV-visible spectroscopy, fluorescence spectroscopy, FT-IR spectroscopy, Raman spectroscopy, scanning electron microscopy (SEM), transmission electron microscopy (TEM), and X-ray photoelectron spectroscopy (XPS) were used to characterize the designed nanomaterial. This nanomaterial exhibited excellent pH sensing properties and showed long term stability (up to one month) without loss of sensor performance.

## 1. Introduction

In the past few decades, there has been increased interest in synthesizing chiral-conducting polymers because of their potential applications as surface-modified electrodes [1] and separation materials [2]. The latest research on conducting polyaniline (PANI) has been targeted to improve its solubility and further processability. Reprotonation of emeraldine base with a second protonic acid [3] and functionalization of the PANI emeraldine base (PANI-EB) [4] have been reported to improve the solubility in the post-processing step. Previous efforts have also used a surfactant to aid in processing PANI derivatives [5] or to polymerize ring-substituted aniline monomers, which contain different substituents to enhance the solubility, including water solubility [6].

Optically active, conducting polymers are of considerable interest [7], because of their wide application in stereoselective analysis [1,8,9], chiral separations [10], and as chemical and biological sensors [11,12]. Chiral polyanilines can be synthesized using chemical oxidation of a chiral agent [13,14,15,16,17,18,19] or by electrochemical polymerization [20,21,22], chemical vapor phase polymerization [23], a non-green approach, or chiral complexation with the chiral palladium complex [24]; the latter method incorporates chiral anions onto the main chain of PANI as counter-ion dopants through electrostatic interaction or hydrogen bonding. Naturally occurring amino acids can act as a dopant, where the -COOH group provides doping to conducting polymers by hydrogen bonding, which in turn stabilizes the chiroptical properties of optically active PANI [25]. PANI with specific morphologies have drawn increased attention because of their different physical and chemical properties, which broaden the application to various fields, such as chemical sensor and biosensors, controlled release and delivery, light-emitting and electronic devices, *etc.* [26,27]. Recently, discrete morphologies (*i.e.*, flakes, flower, and fibers forms) of PANI have been prepared using naturally occurring chiral amino acids as a soft template [28,29] through the electrostatic interaction and hydrogen bonding between PANI and amino acids. Moreover, enzymatic synthesis of electroconductive biocomposites with the aid of DNA has been reported [30]. Remarkably, conductive PANI helixes can be prepared without a chiral dopant, via self-assembly, where the helixes were synthesized by emulsion polymerization with dodecylbenzenesulfonic acid (DBSA) as the dopant as well as surfactant, and ammonium persulfate (APS) as the oxidant [31].

Optical sensors are based on pH-induced, reversible changes in optical properties (absorbance, reflectance, fluorescence, and refractive index). Absorbance spectroscopy is the simplest and most affordable technique, because most laboratories are equipped with UV-Vis absorption spectrophotometers. The pH sensing layer is the key component in an optical pH sensor, which can be made either by immobilizing a pH-sensitive organic dye in polymer or by a covalent attachment of an organic dye onto the tip of an optical fiber. Nevertheless, covalent bonding requires a long and tedious procedure, which might cause a loss in the dye’s sensitivity and, thereby, lead to poor absorption and fluorescence properties [32]. Because of this deficiency, conducting polymers (polyaniline and polypyrrole) might be promising candidates for serving as optical sensing material without involving an organic dye [33,34].

PANI exists in a de-doped form, known as emeraldine base (EB), which is the most common form of polyaniline. EB undergoes protonation and produces emeraldine salt (ES), also known as the doped form of PANI. The doped form is conductive and electrochemically active and predominates at low pH (pH < 3) [35]. The absorbance of the EB and ES forms of PANI are pH-dependent in the 400–900 nm range. The electronic absorption band of polyaniline that is sensitive to a change in pH is broad and could extend into the near-infrared region [36]. The significant change of EB to the ES form of PANI by protonation has been investigated thoroughly by UV-VIS-NIR spectroscopy [37,38,39]. Doping behavior of PANI with different acid dopants has been explored with the possibility of preparing the polymer of a desired oxidation state [40]. PANI synthesized with a poly(acid) counter-ion, which protonates the insulating EB form, renders PANI films pH-responsive and electroactive [41,42]. The pH-dependent electrochemical properties of chemically and electrochemically polymerized PANI films have also been characterized [43,44,45]. Earlier studies mentioned that polyaniline synthesized under short reaction times (30 min) [37,38] was very stable in water, but it underwent an irreversible color change from blue to yellow after a few hours of exposure to air. Hence, the polyaniline neither retained long-term pH sensitivity nor was suitable for pH sensing. To avoid this problem, Jin *et al.* [34] synthesized stable polyaniline by increasing the polymerization time from 30 min to 12 h [34]. They determined that this increase in reaction time shifted the λ_max_ of PANI from 840 nm to 750 nm, which was attributed to the high oxidation state of polyaniline with a different degree of protonation of the imine nitrogen atoms in the polymer chain [46,47].

In our study, due to its chirality and solubility, we chose (*S*)-naproxen as a dopant to synthesize chiral PANI. The green synthesized chiral PANI was characterized by ultraviolet–visible spectroscopy, fluorescence spectroscopy, Fourier transform infrared (FTIR) spectroscopy, Raman spectroscopy, and X-ray photoelectron spectroscopy (XPS). We studied the morphology of PANI-Nap using scanning electron microscopy (SEM) and transmission electron microscopy (TEM), and we aimed to synthesize highly processable optically-active, nanomaterial, PANI-Nap. The material we synthesized demonstrated better hydrophilicity and long-term stability, which enabled it to function as a fast and stable optical sensor.

## 2. Results and Discussion

### 2.1. Characterization

**Scheme 1:**
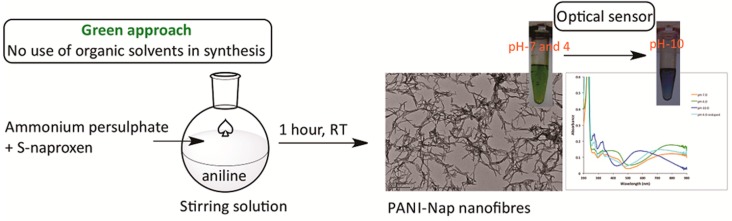
Green synthesis of nano fiber and its application in pH sensing.

**Figure 1:**
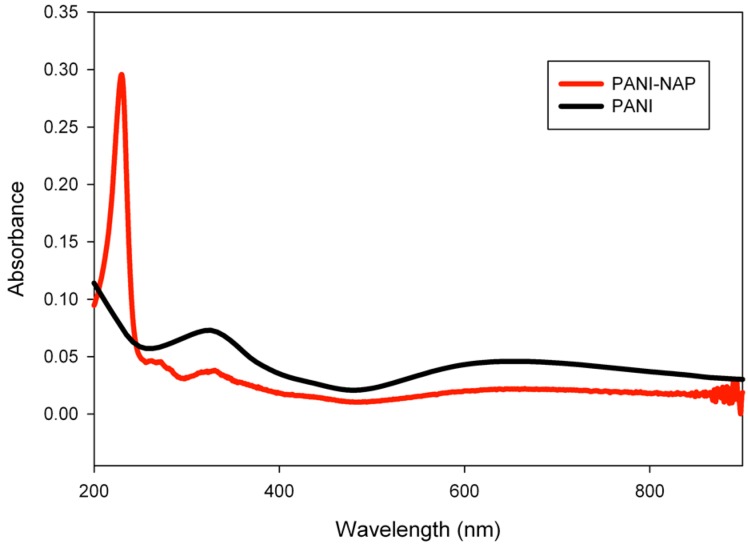
Absorption spectra of PANI and PANI-Nap.

Polymerization of aniline initiated by adding (*S*)-naproxen and ammonium persulfate to a stirring solution of aniline. The reaction was continued for 1 h to complete polymerization process. After that, the solution was centrifuged, washed with deionized water and ethanol to remove excess dopant and monomeric species. The material was redissolved in deionized water to perform the sensing application (a schematic diagram was shown in Scheme 1). After centrifugation and removal of excess dopant and monomeric species by washing, the polymeric material was redissolved in deionized water and studied by UV-Visible and fluorescence spectroscopy. The absorption spectra of PANI and PANI-Nap are shown in Figure 1; the polaron bands at 320 nm was ascribed to π-π* transition of the benzenoid ring. PANI-Nap exhibited an absorption maximum at 230 nm, which represents the doping with (*S*)-naproxen, because naproxen absorbs at this wavelength [48].

**Figure 2:**
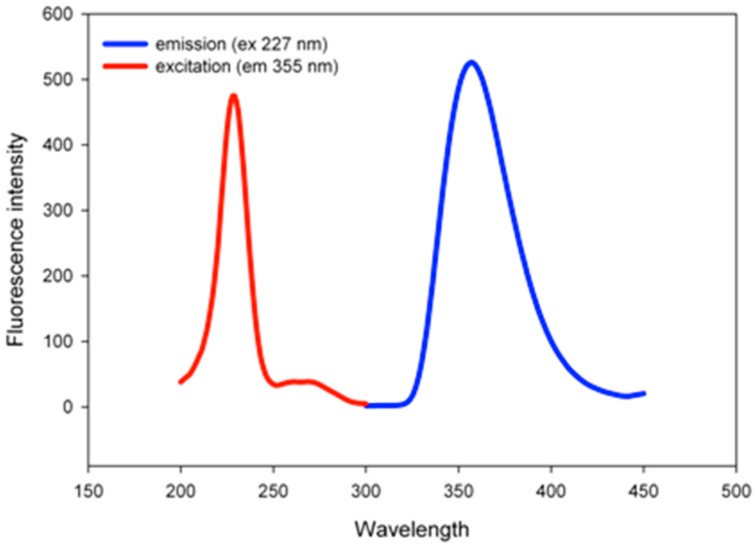
Excitation and emission spectra of PANI-Nap.

Because the naproxen dopant is a fluorescent molecule, it was advisable to confirm the doping of PANI-Nap using fluorescence spectroscopy. A solution of as-prepared PANI-Nap showed maximum excitation and emission at 227 nm and 355 nm, respectively, with a large stoke shift of 128 nm (Figure 2); the emission peak at 355 nm is a characteristic emission peak of naproxen [48], which again confirms that PANI was doped with naproxen.

**Figure 3:**
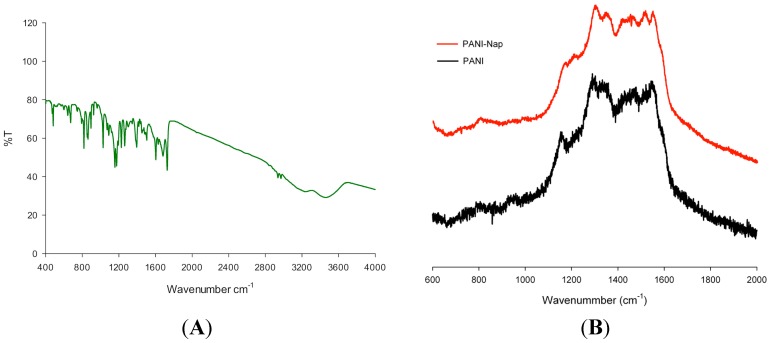
(**A**) FTIR spectra of PANI-Nap; (**B**) Raman spectra of PANI and PANI-Nap.

The FT-IR and Raman (Figure 3A,B) spectral characteristics of PANI-Nap resembled the recently introduced gold nanocomposite, Au-PANI-Nap [49], which was developed in our laboratory. The characteristic bands of PANI at wavenumbers of 1120 cm^−1^ (N=Q=N, Q representing the quinoid ring), 1296 cm^−1^ (assigned to C–N stretching mode), 1495 cm^−1^ (assigned to C=C stretching of benzenoid rings), and 1570 cm^−1^ (assigned to the stretching of the quinoid rings) were observed in the FT-IR spectra and were similar to the emeraldine salt form of PANI [50]. The peaks at 3257 cm^−1^ and 3460 cm^−1^ were assigned to hydogen bonded –OH and –NH stretching, respectively. Peaks at 2957 and 2942 cm^−1^ corresponded to asymmetric and symmetric stretching vibrations of –CH_3_, while the bands at 1629, 1607, and 1480 cm^−1^ corresponded to the stretching vibrations of the aromatic ring observed in PANI-Nap. Bands at 1022 and 865 cm^−1^ corresponded to the absorption of C–O–C in naproxen, while 1173 cm^−1^ was due to the absorption of C–O. The main peaks in the range of 500–1750 cm^−1^ showed a similar pattern to that of native naproxen [51], which suggested that the polymer was doped with naproxen.

The Raman spectra exhibited peaks at 1625, 1596, and 1570 cm^−1^, which represent the characteristic bands of semi-quinone rings. The peak at 1511 cm^−1^ corresponded to the N–H bending deformation band of the protonated amine. Peaks at 1348 and 1252 cm^−1^ were assigned to the C–N stretching modes and the peak at 1179 cm^−1^ corresponded to the C–H bending deformation.

**Figure 4:**
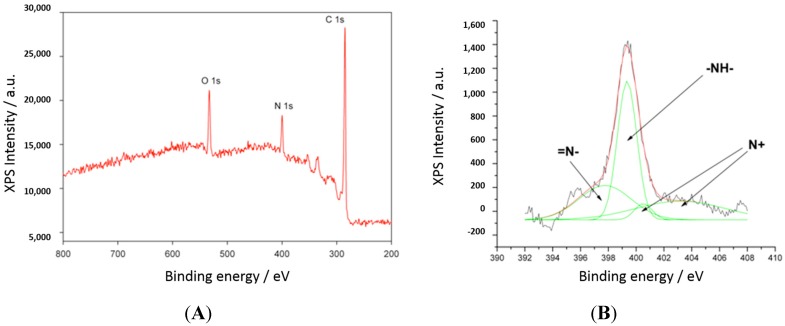
XPS spectra of (**A**) PANI-Nap and (**B**) nitrogen 1s region.

The elemental composition of PANI-Nap was determined using a XPS quantitative spectroscopic technique, which confirmed the presence of C, N, and O in the polymer (Figure 4A). The broad peak in the high-resolution spectra of the nitrogen 1s region (Figure 4B) suggested the presence of several oxidation states of nitrogen. The observed spectrum could be fitted and the resulting peaks have matching assignments reported previously for doped polyaniline [52]. The peaks at 397.5 eV and 399.1 eV were assigned to imine-like (=N–) and amine-like (–NH–), respectively, while the peaks at >400.4 eV and >402.2 eV corresponded to positively charged nitrogen atoms. The presence of electron deficient nitrogen species may be attributed to delocalization of electron density from the polyaniline ring associated with the formation of polaron and bipolaron bands [53].

**Figure 5:**
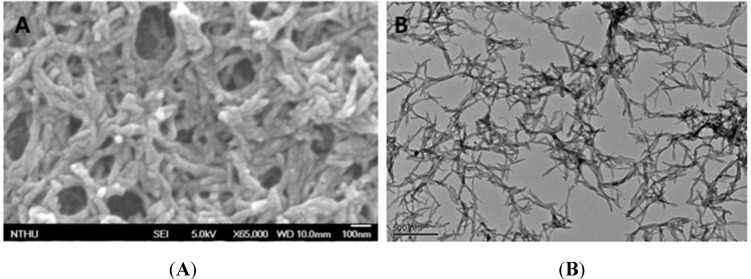
SEM (Scale bar 100 nm, **A**) and TEM images (Scale bar 500 nm, **B**) of PANI-Nap nanomaterial.

The morphology of PANI-Nap was examined by scanning electron microscope and transmission electron microscopy. Figure 5A,B shows the nanofibric morphology of PANI-Nap obtained by SEM and TEM, respectively; the morphology was found similar to the chiral PANI synthesized with camphor sulfonic acid [12,14,54], but different from chiral polyaniline synthesized with l-phenylalanine, where flaky, spherical and urchin-like morphologies were observed [29].

### 2.2. pH Sensing Application

**Figure 6:**
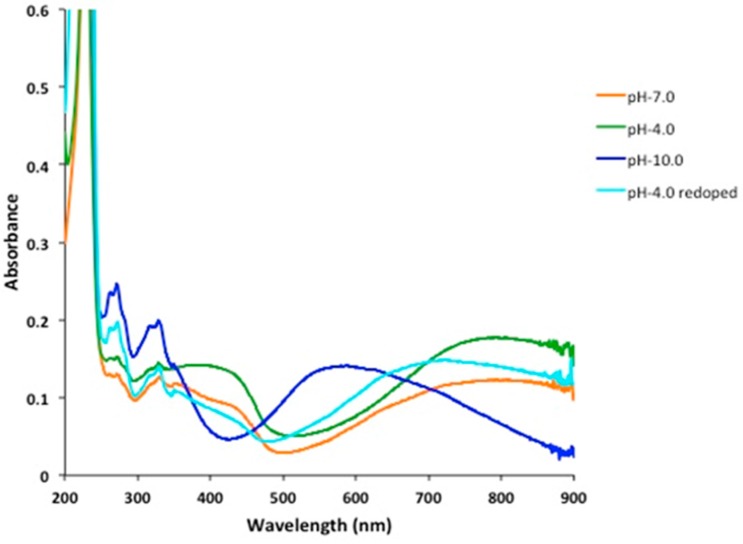
Absorbance responses of PANI-Nap at different pH.

**Figure 7:**
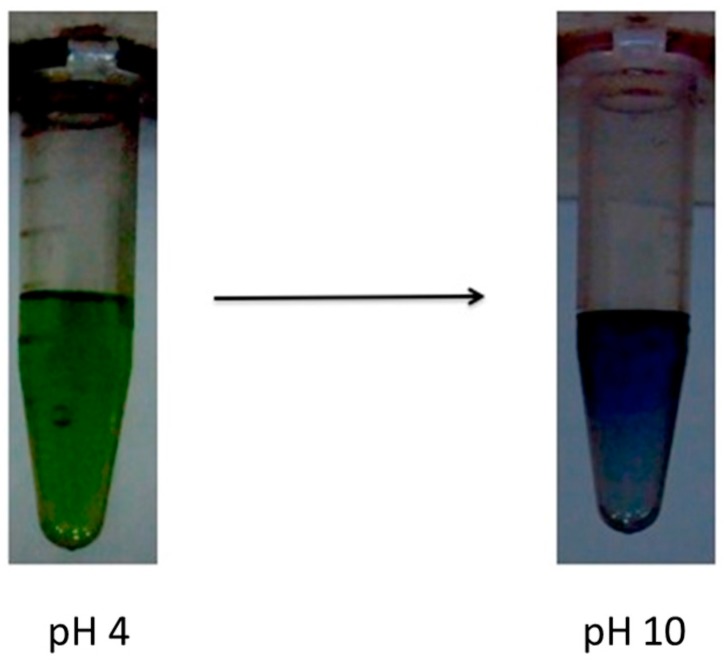
Color change of polymer with respect to pH; polymer exist in green color at pH 4 and 7 while it turns blue at pH 10.

**Figure 8:**
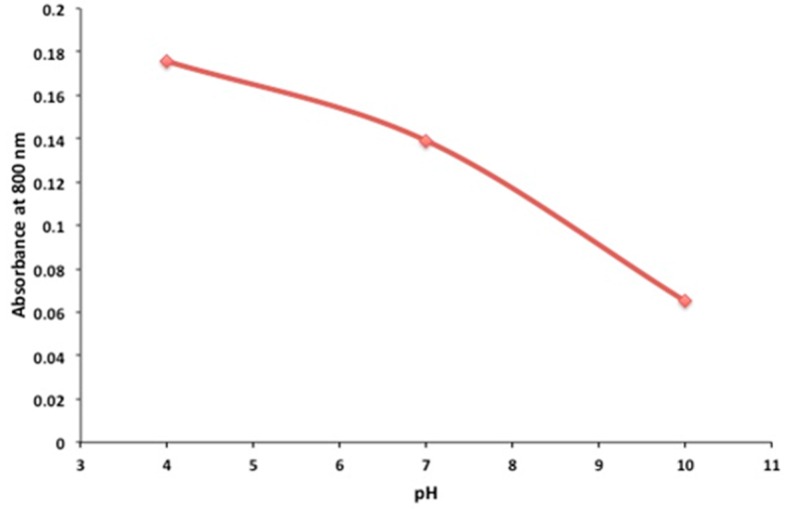
Absorbance change with pH at 800 nm.

**Figure 9:**
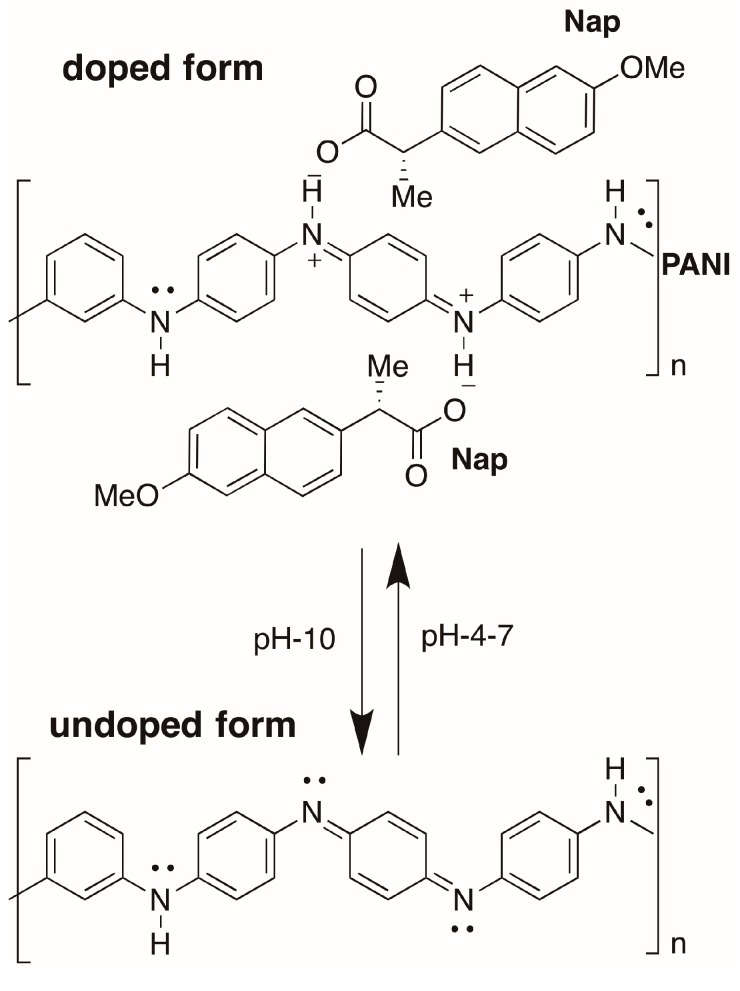
Doped and un-doped form of PANI-Nap.

In view of the current state of research, it was our primary goal to prepare a stable pH-sensitive PANI. The pH-dependence of PANI-Nap was studied by UV-absorption spectroscopy (Figure 6). At pH 4.0, the as-prepared material solution was green in color and existed in its doped form; the peak at 420 nm was assigned to the polaron band of the benzenoid ring, whereas the peak at 320 nm was attributed to the π-π* transition of the benzenoid ring. At pH 10.0, we observed a sharp color change of the solution from green to blue with a decrease and subsequent disappearance of the polaron bands at 420, 750, and higher (750–800) as shown in Figure 7. A plot of the change in absorbance at 800 nm *vs.* pH has been shown in Figure 8. The response time of the optical sensor is 5 s. This was due to the un-doped form of PANI and was probably due to a polymer chain with extended conjugation (Figure 9). Additionally, the new band at 580 nm was attributed to the exciton transition of the quinoid rings. The synthetic method presented herein is facile and environmentally friendly compared to existing methods, because a 1 h reaction time was enough to produce stable, pH-responsive, PANI nanofibers The as-prepared PANI-Nap material can be reproducibly synthesized, and the solution showed long term stability (1 month) without any loss in sensor performance. Moreover, the nanomaterial we prepared was doped with a chiral dopant (*S*)-naproxen, which possesses a unique optical activity, so that the material attracts further attention as a sensing application of chiral compounds, which will be the main focus of our future work.

## 3. Experimental Section

### 3.1. Chemicals and Equipment

Aniline, (*S*)-naproxen, ammonium persulphate and all other chemicals were purchased from Sigma-Aldrich (St. Louis, MO, USA) and they were used without further purification. We used deionized water having a resistivity of not less than 18 M·cm^−1^ (Milli-Q, Bedford, MA, USA) throughout the experiment. UV-Vis and FTIR spectra were recorded using Cary 300 Bio (Varian, Palo Alto, CA, USA) and Spectrum One B (Perkin-Elmer, Waltham, MA, USA) spectrometers, respectively. Surface morphology was characterized using SEM and TEM (JEOL, JEM-1400, Tokyo, Japan). Quantitative analyses were performed using XPS (ULVACPHI XPS spectrometer PHI Quantera SXM, Chigasaki, Japan) and EDX (JEOL JSM-7000F, Tokyo, Japan). A HORIBA JOBIN YVON, HR-800 instrument (Horiba, Kyoto, Japan) was used to measure Raman spectra.

### 3.2. Synthesis and Characterization of PANI-NAP

We added (*S*)-naproxen (200 μL, 2% aq. suspension) and ammonium persulfate (6 mg in 2 mL, 1 M HCl) to a stirring solution of aniline (2 mL prepared by diluting 2 µL aniline with 1 M HCl), and allowed the reaction to proceed for 1 h. After the polymerization, the solution was centrifuged at 20,000 rpm for 15 min. The residue was washed 2–3 times with distilled, deionized water and ethanol to remove excess dopant and monomeric species. The material was dried under vacuum and subsequently used for characterization. Similarly, PANI was also synthesized in the absence of (*S*)-naproxen. At the end of synthesis process, the removal of the monomer, additives and dopants was done by an addition of ethanol and water, followed by centrifugation. The supernatant was discarded and Milli Q water was added to the material to acquire the absorption spectra by UV-Vis spectrophotometer. Another portion of the synthesized material was kept as dried solid for characterization using FT-IR, Raman and XPS. The as-prepared solution and solid were further tested for their stability.

## 4. Conclusions

We, in this study, designed and introduced a novel chiral polyaniline optical sensor. PANI-Nap was synthesized by a rapid and facile one-pot procedure using an environmentally-friendly approach, and it was satisfactorily characterized by various spectroscopic techniques (*i.e.*, UV-visible spectroscopy, fluorescence spectroscopy, FT-IR spectroscopy, Raman spectroscopy). The morphology and additional optical properties of PANI have also been discussed. The as-prepared nanomaterial showed a fast response (5 s) with a change in pH without any change in sensitivity, even after exposure to air. In addition to the excellent optical sensing properties, it showed long term stability (up to 1 month) without loss in sensor performance. Since the material was synthesized using a chiral dopant, we will explore the further applicability of PANI-Nap, especially in the sensing of chiral compounds.
